# Imaging Brain Metabolism and Pathology in Alzheimer’s Disease with Positron Emission Tomography

**DOI:** 10.4172/2161-0460.1000143

**Published:** 2014-04

**Authors:** S Shokouhi, D Claassen, WR Riddle

**Affiliations:** 1 Department of Radiology and Radiological Sciences, Vanderbilt University, Nashville, TN, USA; 2 Department of Neurology, Vanderbilt University, Nashville, TN, USA

**Keywords:** Positron emission tomography, FDG-PET, Alzheimer’s disease, Mild cognitive impairment, Amyloid-beta plaques, Neurofibrillary tangles, PET radiotracer

## Abstract

Current Positron Emission Tomography (PET) biomarkers for Alzheimer’s disease (AD) assess either neuronal function, or associated pathological features of this common neurodegenerative disease. The most widely accepted clinical PET tool for AD is 18-fluorodeoxyglucose PET (FDG-PET), which measures cerebral metabolic glucose utilization rate (CMRglc). FDG-PET is a marker of synaptic activity, neuronal function, and neuronal metabolic activity. AD is characterized by a distinct pattern of hypometabolism, as seen with the FDG images. This pattern can show variability across different subjects and is present before a patient is demented, specifically in amnestic mild cognitive impairment a clinical diagnosis defined as an intermediate state from normal aging to dementia. In addition to FDG PET, novel PET approaches assess known pathological hallmarks of AD including extracellular amyloid-beta plaques (Aβ) and intracellular neurofibrillary tangles composed of tau fibrils. Already, amyloid PET imaging is a tool that allows *in vivo* imaging of extracellular beta-amyloid levels. Efforts to bring tau imaging into clinical use continue, but this approach is hampered by the intracellular nature of tau protein deposition, subsequent weak radiotracer binding, and low image contrast. Several new candidate probes for tau-specific PET imaging are currently available but have not found their way into broad clinical applications.

This study gives an overview of the most recent PET-based neuroimaging techniques for AD. We place special emphasis on PET data analysis and interpretation techniques, as well as radiochemistry for imaging metabolism and assessing Aβ and tau pathology.

## Alzheimer’s Disease and Metabolism

Current Positron Emission Tomography (PET) biomarkers for Alzheimer’s disease (AD) assess either neuronal function, or associated pathological features of this common neurodegenerative disease. 18-fluorodeoxyglucose PET (FDG-PET) is used as a clinical tool providing important complimentary diagnostic information in the assessment of patients with a range of cognitive symptoms. FDG-PET is a marker of synaptic activity, neuronal function, and neuronal metabolic activity [1]. In AD, predominant patterns of hypometabolism are observed in the posterior cingulate cortex, temporal-parietal regions (sometimes asymmetric) and later in the disease course, in the frontal lobes [2–4]. Early on, the posterior cingulate cortex and the neighboring precuneus are most commonly involved. This pattern can show variability across different subjects and is present before a patient is demented, specifically in amnestic mild cognitive impairment [5,6], a clinical diagnosis defined as an intermediate state from normal aging to dementia [7]. The diagnostic accuracy of FDG-PET is frequently defined and discussed in terms of sensitivity (true positive) and specificity (true negative). Clinical assessment or pathologic confirmation can serve as a reference standard for FDG-PET evaluation. Most existing studies compare FDG-PET findings to clinical assessments. Although there are several specific criteria used in the clinical diagnosis of AD, there is no single reliable clinical test and a definitive diagnosis is only possible through post-mortem observation of amyloid-beta plaques and neurofibrillary tangles. Bohnen et al. [8] reported that an average diagnostic accuracy of 93% (96% sensitivity and 90% specificity) can be expected to differentiate AD subjects from healthy controls using FDG-PET cross-sectional case-control study with clinical assessment as reference standard. Temporal relationships between cognitive decline and metabolic changes can also be assessed with FDG-PET. Recent longitudinal FDG-PET studies [9,10] have shown associations between metabolic changes and cognitive tests, such as the Alzheimer’s Disease Assessment Scale’s cognitive subscale (ADAS_cog) [11], the Mini Mental Status Examination (MMSE) [12] and the Functional Activities Questionnaire (FAQ) [13]. Furthermore, FDG-PET has been utilized for differential diagnosis of AD versus other forms of dementia, such as the dementia with Lewy bodies (DLB), the second most frequent type of dementia. Consistent hypometabolism in medial occipital cortex were observed in DLB but not in AD suggesting the use of FDG-PET as a diagnostic aid to differentiate DLB from AD [14,15]. The simplest form of FDG-PET evaluation involves qualitative interpretation of images by a clinician to find areas that show abnormal metabolic activity. The quality of the diagnosis depends on the training and experience of the observer. Semi-quantitative methods, such as the standardized uptake value ratio (SUVR) calculate the normalized mean FDG activity of selected regions using cerebellum as reference region for normalization. In recent years, several software packages have become available that allow a voxel-based statistical analysis of FDG-PET data to aid the clinical interpretation of AD and MCI cases [16,17]. Some of these programs are directly provided by the vendors of PET scanners. Others, such as Neurostat, can be freely downloaded (*128.95.65.28*/~Download/) and installed on a personal computer. This technique involves the spatial normalization and smoothing of each subject’s PET scan to a reference brain followed by a voxel-by-voxel statistical comparison of the FDG activity against the mean and standard deviation of an age-matched control database to obtain the so-called z scores and generate 3D surface-extracted images of hypometabolic areas. While the voxel-based statistical analysis provides an observer-independent outcome, it could have potential limitations. The analysis is performed in a standard reference brain volume and there are interpolation effects due to spatial normalization [18]. Furthermore, the voxel-based parametric mapping relies on comparisons to a control population, which could be contaminated by individuals with pre-symptomatic AD [19]. The sample size of the control population can affect the diagnostic performance and possible mismatches in PET scanners/image reconstruction between control population and patients can create bias [20]. While these issues seem to be less critical to the diagnostic accuracy of FDG-PET for AD or MCI, they can become more important as we move towards the pre-symptomatic stages of the disease. AD is heterogeneous disease with variable progression rate of hypometabolism [21] and in the manifestation of cognitive features [22]. It is not known to what extent the incipient pre-symptomatic phase of Alzheimer’s disease causes metabolic changes. If there are subtle AD-related metabolic abnormalities prior to the onset of mild cognitive impairment, methods that are less sensitive to metabolic heterogeneities across subjects are needed to detect them. Habeck et al. [23] introduced multivariate approaches to evaluate correlation/covariance of FDG activity measures across brain regions to identify metabolic connectivity networks in the brain as a sensitive marker for capturing subtle metabolic disruptions. Lee et al. [24] showed the implementation of a voxel-wise interregional correlation analysis on FDG-PET as a robust tool for highlighting resting state metabolic connectivity. In this study, anatomical regions were used as seed volumes of interest and the results showed characteristic patterns of connectivity throughout the lobes, gyri and Brodman areas, which were independent of the size of the seed volumes or the method used to define them. Established diagnostic application of metabolic networks with FDG-PET is also found in Parkinson’s disease (PD) [25] where the abnormal and reproducible disruptions of metabolic networks show correlations with the clinical progression of the disease (termed as PD-related motor and cognitive metabolic covariance patterns). Morbelli et al. [26] utilized a voxel-based statistical approach to define clusters with significant metabolic differences between two subject groups. In the first comparison, these two groups were the education-matched amnestic MCI (who later converted to AD) patients and the healthy controls. Then the metabolism was compared between highly and poorly educated amnestic MCI patients. The clusters of significant depression and compensation were further used as volumes of interest in a brain interregional correlation analysis to explore metabolic connectivity and the impact of education in compensatory networks. Since FDG-PET typically provides one image per subject, the metabolic connectivity was explored at group level only. However, more recent studies [27,28] have demonstrated the feasibility of FDG-PET based connectivity analysis at the level of individual subjects. Di et al. [29] had hypothesized that functionally connected regions would also show higher metabolic correlations. To prove this hypothesis, they performed two types of connectivity analysis on both functional MRI (fMRI) and FDG-PET. While the results demonstrated that the FDG-based metabolic networks were similar to the resting-state functional networks (mainly homotopic networks), there were also some discrepancies. Several factors, such as the tissue characteristics, metabolism, cerebral blood flow (CBF) and cerebral blood volume (CBV) were considered to impact the neural activity and perhaps all together shape the underlying neural network architectures seen in fMRI. Therefore, it is important to obtain connectivity maps from different imaging techniques and see how they each change during the course of a disease. Yakushev et al. [30] examined the metabolic and structural connectivity (diffusion tensor imaging) in relation to normal working memory. Due to its low temporal resolution, FDG-PET captures steady state neuronal activity independent of vascular factors. In another recent study [10], we measured the temporal correlation between each subject’s regional FDG distribution at baseline and follow-up scans as an FDG analysis method with less sensitivity to metabolic heterogeneities across populations. For each subject, the correlation data were plotted as a function of time and compared to the subject’s changes in cognitive scores. The results not only demonstrated a faster temporal correlation decline in individuals at AD risk, such as those with APOE-e4 allele and mild cognitive impairment, but also established a direct association between visit-to-visit changes in the temporal FDG-PET correlations and visit-to-visit changes in cognitive test scores within individual subjects. Multivariate techniques that assess within subject spatial and temporal correlations could become increasingly important methods in capturing subtle and heterogeneous disruptions in the brain metabolic map and possibly help identifying pre-symptomatic AD candidates among the healthy population. In addition, the emerging field of multi-modal Positron Emission Tomography with Magnetic Resonance Imaging, PET/MRI, allows simultaneous acquisition with both modalities and serve as a useful tool to compare FDG-PET with fMRI to provide comprehensive and complementary information about the brain function, similar to previous preclinical small-animal studies [31]. FDG-PET could also be compared with the arterial spin labelling MRI (ASL). Musiek et al. [32] made a comparison between these two modalities by injecting FDG during the ASL acquisition and performing PET imaging 40 minutes later.

## Alzheimer’s Disease and Aβ Plaques

Neuroimaging techniques for Aβ plaques have been used for the differential diagnosis of AD [33–35] due to the moderate to severe presence of plaques in every AD patient, which possibly develop years before the onset of memory decline and conversion to AD [36,37]. In contrast, many other forms of dementia, such as vascular dementia (VaD) or frontotemporal dementia (FTLD) are not accompanied by Aβ plaques. There is a great demand for assessing the amyloid burden at earlier stages where trial therapies are more effective. Aβ plaques have been imaged using PET with several radiotracers. Pittsburgh compound B [38], ^11^C-PiB, is the first and most extensively examined PET maker of Aβ with several favorable key properties. The lipophilic thioflavin-T derivative molecular structure of ^11^C-PiB crosses the blood-brain barrier and binds with high affinity and specificity to the plaques. The non-specific clearance is fast allowing imaging at high contrast within a sufficiently long time window. Previous ^11^C-PiB studies in human AD patients have shown increased cortical binding of ^11^C-PiB in AD subjects compared to normal controls (60%–70%) [39,40]. The overlapped patterns of ^11^C-PiB uptake and the regional distribution of Aβ plaques were verified via post-mortem immunohistochemistry revealing that the binding was more prominent in areas known to have high Aβ deposit such as the tempo-parietal and frontal regions. In contrast, modest tracer uptake was observed in the cerebellum. Despite optimal kinetic properties of ^11^C-PIB, the 20 minute half-life of the labelling ^11^C isotope is a limiting factor for applications in most research centers that are not in close vicinity of a cyclotron (a particle accelerator that generates ^11^C isotopes by colliding high-speed moving charged particles to a target material). As an alternative, ^18^F-labled radiotracers allow for a broader clinical use due to their longer half-life of 110 minutes. Several ^18^F labeled tracers are currently available. ^18^F-Florbetapir (^18^F-AV-45) [41,42] has successfully replicated the imaging data obtained with ^11^C-PIB finding wide use as a research biomarker in the Alzheimer’s Disease Neuroimaging Initiative (ADNI) and other clinical trials. Based on a phase-3 multicenter study with ^18^F-Florbetapir, the PET imaging outcomes demonstrated high correlation with the postmortem Aβ distribution [43]. ^18^F-BAY94-9172 also known as ^18^F-florbetaben is another ^18^F-labelled amyloid PET tracer tested on human subjects in 2008 [44]. ^18^F-florbetaben data show 53% increase in the neocortical SUVR in AD subjects compared to the healthy controls, slightly less than ^11^C-PIB, although the cortical distribution of the two tracers are almost identical. In another study [35], the ^18^F-florbetaben cortical uptake was compared between AD and DLB, which is also characterized by the presence of Aβ pathology and the outcomes indicated lower global ^18^F-florbetaben binding in DLB patients, which was different than the outcomes of previous ^11^C-PIB studies in DLB [45,46]. Nevertheless, this study showed the feasibility of 18F-florbetaben in differential diagnosis between AD, MCI, DLB, FTLD, VaD and Parkinson’s disease. ^18^F-FDDNP is another amyloid PET radiotracer that also binds weakly to neurofibrillary tangles [47]. Compared to healthy controls, ^18^F-FDDNP shows only modest increase in cortical binding of AD patients, which reduces the diagnostic value based on visual assessment [48]. Other, quantitative comparisons between ^11^C-PiB with ^18^F-FDDNP in AD, MCI and healthy controls demonstrated that differences in binding potential between the three groups were more pronounced for ^11^C-PiB than for ^18^F-FDDNP [49,50]. ^18^F-flutometamol [51,52], which is structurally identical to ^11^C-PiB, and ^18^F-AZD4694 [53] are among the other most notable ^18^F-lableled amyloid PET tracers. Both ^18^F-flutometamol and ^18^F-Florbetapir are approved for selective clinical use by the US food and Drug Administration (FDA). While more lipophilic radioligands will display faster accumulation in the brain than less lipophilic ones, if the radiotracer is too lipophilic, it will be bound by plasma proteins and undergo fast metabolism by enzymes [54]. Radiolabeled metabolites can bind to a different target, thus displaying a higher non-specific uptake. Defluorination is one example for metabolism specifically observed in ^18^F-labeled radiotracers. All ^18^F-labeled tracers have high non-specific white matter uptake often distinctively visualized in their PET images of healthy subjects [55]. In AD subjects, most ^18^F-labeled tracers show a higher non-specific background than ^11^C-PiB. The ratios of frontal cortex to white matter for ^11^C-PiB are 0.8, 1.1, and 1.3 in controls, MCI, and AD [40]. These ratios are lower for all other ^18^F-labelled radiotracers, such as ^18^F-florbetaben (0.7, 0.8, and 1.1), ^18^F-flutemetamol (0.4, 0.5, and 0.7) [52] and ^18^F-florbetapir (0.7 and 1.1) for controls and AD [42]. While it is important that a radiotracer efficiently crosses the blood brain barrier and binds to the plaques at high affinity and specificity, the most critical and difficult to develop property is the non-specific clearance. It is defined as the speed with which the radiotracer is washed out from the brain regions that don’t have amyloid plaques. This process reduces the non-specific background down to an acceptable level to allow imaging at a sufficient contrast-to-noise ratio. Due to the short half-lives of the radioactive isotopes, there is a small margin of time to wait for the non-specific clearance and the scanning process should start no later than 30–90 minutes after the injection (Chester Mathis, leading radiochemist in ^11^C-PiB development). Most clinical Aβ-PET data analyses published to date utilize the cortical-to-reference ratios, also known as standardized uptake value ratio (SUVR) [55–57] due to its computational simplicity and shorter scan time. The radiotracer uptake is measured after bindings in cortex and the reference region reach a steady state post injection. The SUVR threshold for normal binding is 1.5. Any ratios above this threshold are referred to as PIB-positive and indicate abnormal accumulation of dense plaques. Alternatively, kinetic modeling of amyloid PET data can be calculated from dynamic data [58,59]. This process requires a longer scan time and is more vulnerable towards subject movement during the scan. The relationship between regional brain uptake of PET amyloid radiotracers and cognition has been the focus of several studies. Using ^11^C-PiB and ^18^F-flutemetamol as radiotracer, the Australian research team led by Christopher Rowe [60] showed that amyloid-negative healthy older adults had no change in episodic memory or any other aspects of their cognitive function. Similarly, amyloid-negative older adults with MCI had no further decline in memory or other cognitive domains, which together with other previous studies [61] supported the hypothesis that individuals diagnosed with MCI but with no amyloid-positive scan most likely will not convert to AD. The same study found that amyloid-positive healthy adults also declined moderately in memory but not in other cognitive domains when compared to amyloid-negative healthy adults. Compared to amyloid-negative healthy controls, amyloid-positive MCI subjects had greater decline in both memory and in the same magnitude in other cognitive functions, such as attention and language. The AD group with amyloid-positive scan showed large decline in all cognitive functions, particularly in language and visuospatial domains. While previous PIB-PET have shown that Aβ plaques can accumulate years before the clinical onset of the disease, there is no strong correlation between the magnitude of Aβ accumulation and the severity of cognitive functions. However, there is a strong association between the rate of the Aβ accumulation and the rate of the cognitive decline [60,61]. Jack and colleagues suggested that the rate of amyloid accumulation slows and reaches a plateau with the clinical manifestation of AD [57, 62] and proposed a model that related each stage of the disease to a different biomarker. According to this model, Aβ accumulation becomes abnormal in the initial pre-symptomatic phase. As the disease progresses, the Aβ accumulation rate slows down, abnormal metabolic and structural changes become more prominent and these correlate with the severity of clinical symptoms. Therefore, longitudinal Aβ-PET studies are particularly important and the impact of the methodology for the analysis of longitudinal amyloid PET should be investigated more thoroughly. While a few recent studies have compared the outcomes of several existing methods of longitudinal amyloid-PET analysis [63], it is essential to explore more sophisticated neuroimaging data analysis techniques to follow the slow and protracted progression of Aβ deposits [64]. Most current methods rely on the mean/median of the voxel activity values across a region of interest (ROI) without accounting for the spatial activity distribution within the region. It is conceivable that the estimation of subtle changes is impended by the presence of noise in amyloid-PET images, thus the regional mean values are associated with high variance in the voxel activity. There still exists a need for longitudinal amyloid-PET image analysis methods that can model stochastic relationships between voxel values within a region and incorporate this information into an image analysis framework to distinguish whether the changes in the spatial distribution of voxel activities are attributed to the biological effects (progression of Aβ accumulation) or to image noise.

## Alzheimer’s Disease and Neurofibrillary Tangles

Neurofibrillary tangles composed of tau fibrils (NFTs) are an increasingly recognized part of the AD pathogenic process. Their intracellular formation around nerve endings stands in contrast to the extracellular accumulation of Aβ plaques. The fibrils of tau in AD brains are termed paired helical filament (PHFs), a structural form that tau proteins seem to aggregate in AD [64]. In AD the tau protein is surrounded by the more abundantly present Aβ protein. It is not easy to generate tau ligands without affinity for amyloid. A useful tau ligand should have a high tau–Binding potential/amyloid-Binding potential ratio. Most of the past research efforts for developing tau ligands were focused on PHF-tau. Okamura et al. [65] screened over 2000 molecules to identify those that could cross the blood brain barrier and the cell membrane and bind with high affinity and specificity to intracellular tau aggregates. They introduced three quinoline and benzimidazole derivatives (BF-126, BF-158, BF-170) as the first candidate probes. These led to the synthesis of the first generation of THK series, the ^18^F-THK523 [66] followed by the second generation probes including ^18^F-THK-5105/^18^F-THK-5117 [67] with enhanced pharmacokinetics and binding characteristics. Zhang et al. [68] tested over 900 compounds using autoradiographic assays on human brain tissue sections and introduced the ^18^F-T807/^18^F-T808 probes. Chien and colleagues introduced the first human brain images with ^18^F-T807 [69] and in more recent study with ^18^F-T808 [70], which showed slower 18F-deflourination and faster pharmacokinetics than ^18^F-T807. Most of the current tau probes bind to tau depositions in non-AD tauopathy brains without Aβ plaque depositions, which include a large group of other neurodegenerative diseases. Due to the broad spectrum of tau aggregates, the binding characteristic of tau tracers is affected by different conformations of tau structures. Expanding the binding ability of a radiotracer to a wider range of tau aggregates would allow a more exclusive investigation of tauopathy in both AD and non-AD patients as well as in transgenic mouse models that could develop different forms of tau aggregates [71]. Recently, a new class of tau ligands, the phenyl/pyridinyl-butadienyl-benzothiazoles/benzothiazoliums (PBBs), were developed that were selective for a broader range of tau structures [71]. As a part of this study, a subset of the PBBS, ^11^C-PBB2 and ^11^C-PBB3 were radiolabelled for PET imaging of transgenic mouse models of tau pathology. ^11^C-PBB3 was selected as the candidate with the most prominent visualization of tau lesions in mice. ^11^C-PBB3 was subsequently used for a human PET study with AD patients, normal controls and patients with probable cortico-basal degeneration, a non-AD neurodegenerative disease that is associated with the presence of tau lesions. In addition to ^11^C-PBB3, all subjects were imaged with ^11^C-PiB which confirmed the good affinity of ^11^C-PBB3 for tau aggregates but not for amyloid beta. The scanner resolution is another challenging factor for tau imaging with PET [72]. While partial volume effect is more or less a general problem in PET, it can be more prominent in tau imaging due to the inherent anatomy of tau causing weak radiotracer binding and low image contrast. The AD diagnostic field has long awaited a validated tau tracer. The recent developments of tau-specific PET radiotracers emphasize the unique position of positron emission tomography as a powerful tool for molecular imaging of two major pathologies related to Alzheimer’s disease. All current sets of tau ligands were tested in a small number of human subjects. Similar to Aβ tracers, the current tau tracers will have to undergo a rigorous validation before regular use in the clinic. The longitudinal progression pattern of tau is different than the longitudinal progression of amyloid [73]. The severity of tau accumulation is correlated to the severity of cognitive symptoms [74–77]. Therefore, the diagnostic value of tau imaging could increase in the future to monitor the disease progression and evaluate the response to drugs that target neurofibrillary tangles [77–81]. Tau formations can cause neuronal damages, which lead to the abnormal leakage of Tau in the cerebral spinal fluid (CSF). Several studies have demonstrated a moderate to severe increase of CSF tau in AD. However, elevated CSF tau is also found in other forms of dementia, such as FTD, DLB and sometimes in Parkinson’s disease [82]. While the addition of phosphorylated tau (P-Tau) can increase the specificity of these tests, molecular imaging techniques would provide further important information regarding the spatial distribution of the tau aggregates in brain by showing the regions that are affected and how they spread over time.

In general, the advantage/disadvantage of utilizing a certain AD biomarker would depend on the time point that it is used. Different AD biomarkers have different temporal trajectories as graphically demonstrated by Jack et al. [83] who tracked pathophysiological processes in AD to give an updated dynamic model of five different AD biomarkers. These included FDG-PET, CSF Tau, MRI (hippocampus volume), CSF Aβ42 and Aβ-PET. It is conceivable that the time window at which a biomarker has the highest dynamic and variable behavior is the best time for using that biomarker in the diagnostic work-up of Alzheimer’s disease (Maria Carrillo, Alzheimer’s Association vice president of medical and scientific relations). According to this model, Aβ accumulation in brain precedes cognitive symptoms by years starting with an initial fast accumulation rate that slows over the time and reaches a plateau when dementia is manifested. Changes in CSF tau occur after Aβ accumulation and they can continue while the Aβ has slowed down or plateaued. Therefore, amyloid and Tau imaging would provide complementary information related to the disease progression. Other methods, such as FDG-PET or MRI Hippocampus volume measurements are characterized by later onset and have different trajectories than Aβ and tau. Overall, similar sequential pattern of AD biomarkers were found by other researchers [84–89]. Bateman et al. [88] analyzed longitudinal data from a large cohort of participants using biomarker changes (clinical and cognitive test, brain imaging and CSF) in autosomal dominant AD (ADAD). According to the authors, some of the findings can be transferred to sporadic form of AD (perhaps supporting common pathophysiology between the two forms). However, they also pointed out some differences between ADAD and the sporadic form, such as regional difference in PIB-PET activity. Benzinger et al. [89] expanded Bateman’s analysis to examine trajectories of different biomarkers across the entire brain. They found regional variability and noted the importance of additional research to investigate how biomarker trajectories differ between different subgroups of AD. Based on their longitudinal ^11^C-PIB analysis, Villain et al. [85] identified the existence of two subgroups with respect to Aβ accumulation (“PIB accumulators”, which contain more PIB-positive subjects and the “PIB non-accumulators”). In another study [86], they took a more detailed look into the sequential relationship between brain atrophy and FDG-PET. The findings suggested that the hippocampus atrophy leads to disruption of the white matter tracks (cingulum bundle and uncinate fasciculus) causing hypometabolism in cingulate and subgenual cortices. Förster et al. [87] investigated the relationship between baseline amyloid deposition and subsequent longitudinal changes in FDG-PET hypometabolism and indicated the existence of two different pathological phases of Aβ progression.
